# Prediction of acute kidney injury risk after cardiac surgery: using a hybrid machine learning algorithm

**DOI:** 10.1186/s12911-022-01859-w

**Published:** 2022-05-18

**Authors:** Yelena Petrosyan, Thierry G. Mesana, Louise Y. Sun

**Affiliations:** 1 Cardiocore Big Data Research Unit, University of Ottawa Heart Institute, 40 Ruskin Street, Ottawa, ON K1Y 4W7 Canada; 2 Division of Cardiac Anesthesiology, University of Ottawa Heart Institute, 40 Ruskin Street, Ottawa, ON K1Y 4W7 Canada; 3 School of Epidemiology and Public Health, University of Ottawa, 600 Peter Morand Cres, Ottawa, ON K1G 5Z3 Canada

**Keywords:** Cardiac surgery-associated acute kidney injury, Machine Learning, Random Forests, Data mining, Predictive modeling

## Abstract

**Background:**

Acute kidney injury (AKI) is a serious complication after cardiac surgery. We derived and internally validated a Machine Learning *preoperative* model to predict cardiac surgery-associated AKI of any severity and compared its performance with parametric statistical models.

**Methods:**

We conducted a retrospective study of adult patients who underwent major cardiac surgery requiring cardiopulmonary bypass between November 1st, 2009 and March 31st, 2015. AKI was defined according to the KDIGO criteria as stage 1 or greater, within 7 days of surgery. We randomly split the cohort into derivation and validation datasets. We developed three AKI risk models: (1) a hybrid machine learning (ML) algorithm, using Random Forests for variable selection, followed by high performance logistic regression; (2) a traditional logistic regression model and (3) an enhanced logistic regression model with 500 bootstraps, with backward variable selection. For each model, we assigned risk scores to each of the retained covariate and assessed model discrimination (C statistic) and calibration (Hosmer–Lemeshow goodness-of-fit test) in the validation datasets.

**Results:**

Of 6522 included patients, 1760 (27.0%) developed AKI. The best performance was achieved by the hybrid ML algorithm to predict AKI of any severity. The ML and enhanced statistical models remained robust after internal validation (C statistic = 0.75; Hosmer–Lemeshow *p* = 0.804, and AUC = 0.74, Hosmer–Lemeshow *p* = 0.347, respectively).

**Conclusions:**

We demonstrated that a hybrid ML model provides higher accuracy without sacrificing parsimony, computational efficiency, or interpretability, when compared with parametric statistical models. This score-based model can easily be used at the bedside to identify high-risk patients who may benefit from intensive perioperative monitoring and personalized management strategies.

**Supplementary Information:**

The online version contains supplementary material available at 10.1186/s12911-022-01859-w.

## Background

Acute kidney injury (AKI) is a serious complication after cardiac surgery with an incidence of 5–30% depending upon procedure type and definitions used [1–5]. It is associated with an increased rate of mortality, hospital length of stay, and healthcare cost [6, 7]. As the incidence of AKI is higher after cardiac surgery as compared to medical and noncardiac surgical populations [8], much research has been dedicated to the identification of modifiable risk factors and/or derivation of AKI risk prediction models in this group [9–12].

Recent research demonstrates that there is no standard approach to AKI prediction for patients undergoing cardiac surgery. Existing predictive models are based on different combinations of risk factors and rely heavily on intra- and post-operative events to achieve predictive accuracy [12, 13], while *preoperative* risk stratification is most important and remains challenging. In addition, most existing predictive models were developed to identify patient at risk of severe AKI requiring renal replacement therapy [5, 12], despite mild AKI being associated with up to a threefold increase in the risk of short- and long-term mortality after cardiac surgery [3, 14].

Renal function has long been held as a surrogate for systemic perfusion, and accurate *preoperative* prediction can help to identify patients who may benefit most from intensive monitoring and personalized management strategies throughout the perioperative period. In the advent of artificial intelligence (AI) in medicine, Machine learning (ML) methods such as Random Forests have successfully been applied to create accurate and reliable predictive models in several fields of study [15, 16]. Moreover, hybrid ML algorithms offer improved performance, [17] interpretability and ease of use, making the AI “explainable” to clinicians.

We performed a case study to: (1) derive and internally validate a *preoperative* model to predict AKI of any severity after cardiac surgery, using a hybrid ML approach, consisting of Random Forests, followed by high-performance logistic regression, and (2) compare the performance of this ML model with traditional and enhanced regression models. We hypothesized that the ML model will outperform traditional models, both in terms of performance and parsimony.

## Methods

### Design and selection criteria

The study protocol was approved by the University of Ottawa Heart Institute Research Ethics Board, which waived the requirement for individual patient consent. We conducted a retrospective study of adult patients (age ≥ 18 years) who underwent major cardiac surgery requiring cardiopulmonary bypass between November 1st, 2009 and March 31st, 2015 at the University of Ottawa Heart Institute. Patients who underwent off-pump or thoracic aortic procedures, cardiac transplantation and insertion of ventricular assist devices, as well as those who were dialysis-dependent at baseline, were excluded from the study.

### Data sources

We performed a retrospective analysis of prospectively collected data from Cardiocore. Cardiocore is a multimodular data reservoir that captures detailed demographics, comorbidities, physiologic and procedural details, and perioperative outcomes for all patients who undergo cardiac procedures at the University of Ottawa Heart Institute, a university-affiliated tertiary cardiac care referral center that performs the full scope of cardiac procedures. It is formally managed by a multidisciplinary committee and undergoes regularly scheduled quality assurance audits [18].

### Study outcome

Postoperative AKI was defined according to the Kidney Disease: Improving Global Outcomes (KDIGO) criteria as a serum creatinine increase ≥ 26 μmol/l within 48 h following surgery or an increase of ≥ 50% from baseline within 7 postoperative days [19].

### Candidate variables

We included, a priori, preoperative factors known to be or that could be associated with cardiac surgery-associated AKI based on previous research (Additional file 1: Table S1). Demographic factors included: age [5, 20], sex [11], body mass index (BMI) [20, 21], smoking status [20], and alcoholism status. Preoperative patient characteristics included: glomerular filtration rate (eGFR) [20, 22], preoperative anemia [20, 23], left ventricle ejection fraction [20], Cardiac Anesthesia Risk Evaluation (CARE) mortality risk score [24, 25], a history of atrial fibrillation [9], hypertension [20], coronary artery disease, Canadian Cardiovascular Society (CCS) grading of angina severity [26], recent myocardial infarction within 6 weeks prior to surgery, New York Heart Association Function (NYHA) Class [13], right-sided heart failure, infective endocarditis [27], peripheral arterial disease [20], carotid disease [9], cerebrovascular disease related and unrelated to carotid disease [11], presence of residual neurologic deficit after stroke, seizure disorder, smoking, diabetes [5, 9, 20], preoperative cardiogenic shock [22], preoperative intra-aortic balloon pump therapy and cardiac arrest [5, 22, 27]. Procedure-related characteristics included: operative priority [22, 28], procedure type [13, 20, 22], and redo sternotmy [29].

### Statistical analysis

We divided the cohort randomly into derivation (70%) and validation (30%) samples.

We created three AKI risk prediction models in the derivation samples: (1) a hybrid ML algorithm, consisting of Random Forests, followed by high-performance logistic regression, (2) a traditional statistical model that employed backward variable selection, and (3) an enhanced statistical model that used 500 bootstrap samples for backward variable selection [30]. A data analysis and statistical plan was written and filed with a private entity (institutional review board) before data were accessed.

### Derivation using a hybrid ML algorithm

Details of the Random Forests method have been described elsewhere [31–33]. In short, we used a bootstrap sample of the data to build each of the classification trees. A random subset of variables was selected at each split, thereby constructing a large collection of decision trees with controlled variation. The Random Forests trees are not pruned, so as to obtain low-bias trees (Additional file 2: Figure S1). Every tree in the forest casts a “vote” for the best classification for a given observation, and the class receiving most votes results in the prediction for that specific observation.

The derivation dataset was first sampled to create an in-bag partition—(2/3 of derivation sample) to construct the decision tree, and a smaller our-of bag partition (1/3 of derivation sample) to test the constructed tree to evaluate its performance by computing (Additional file 3: Figure S2): (1) misclassification error, (2) C-statistics, Hosmer–Lemeshow (H–L) *p*-value and (3) model performance (i.e., sensitivity, specificity, positive predictive value [PPV], negative predictive value [NPV]). Then, we performed tenfold cross validation to evaluate the model. The optimal number of trees and a subset of variables at each node was selected using the “tuneRF” package in R (version 3.2.3) to minimize the misclassification error. Random Forests calculates estimates of variable importance for classification using permutation variable importance measure (VIM) [31], which is based on the decrease of a classification accuracy when values of a variable in a node of a tree are permuted randomly. In our cohort, optimal misclassification rate was achieved by using 700 classification trees and 10 variables available for splitting at each tree node.

In this analysis, we converted all categorical variables into a set of binary variables to indicate the absence or presence of a given categorical effect, to increase the computational complexity for tree creation and to mitigate the inherent bias of Random Forests that favors categorical variables with multiple degrees of freedom [34]. We identified a subset of top 30 predictor variables out of the 43 candidate variables and incorporated them into a high-performance logistic model (SAS 9.4, SAS Institute, USA) to identify the best parsimonious model [35]. We used the Schwarz Bayesian Criterion (SBC) as a penalized measure of fit for the logistic regression model to avoid over-fitting [36]. A model with smaller SBC value is preferred over a model with a larger SBC value.

### Derivation using traditional and enhanced statistical approaches

The traditional model employed logistic regression with an automated backward variable selection algorithm and generalized linear model. To prevent overfitting, the association of covariates with postoperative AKI had to have a significance level ≤ 0.001 to remain in the model [37].

The enhanced statistical approach employed backward variable selection for logistic regression models within 500 random bootstrap samples drawn with replacement from the original cohort [30], using a significance level ≤ 0.001 for backward stepwise selection to prevent overfitting [37]. We selected variables that were significant in predicting AKI in 50% or more of the bootstrap samples. We then averaged the regression coefficients for each variable across the 500 bootstrap samples.

### Point score assignment and internal validation

For each of the three models, we assigned integer scores to retained covariates using the method described by Sullivan et al*.* [38] (Additional file 4). We then assessed the discrimination (C statistics or AUC) and calibration (Hosmer–Lemeshow (H–L) goodness-of-fit test and a decile-decile calibration plot of the observed and predicted outcome) of each model using the validation datasets.

The Random Forests analyses were performed in R statistical software (version 3.2.3) using the “randomForest” package [32]. All methods were performed in accordance with the international guidelines for developing and reporting predictive models in biomedical research. The traditional and enhanced statistical models, as well as point score assignment and internal validation, were performed using SAS 9.4 (SAS Institute, USA).

## Results

Of 6522 patients who met the selection criteria, 1760 (27.0%) developed AKI within 7 days of surgery. The baseline characteristics of patients with and without postoperative AKI are reported in Additional file 5: Table S2. These baseline characteristics were similarly distributed across the derivation and validation datasets (Additional file 6: Table S3). Compared to those without AKI, patients who developed AKI were more likely to have undergone complex, emergent surgery, to have higher overall preoperative risk (CARE score ≥ 3), and to have a history of atrial fibrillation, cerebrovascular disease, anemia, and endocarditis.

The crude and adjusted odds ratios representing the relationship between candidate risk factors and AKI are presented in Additional file 7: Table S4.

### Hybrid ML algorithm

**Fig. 1:**
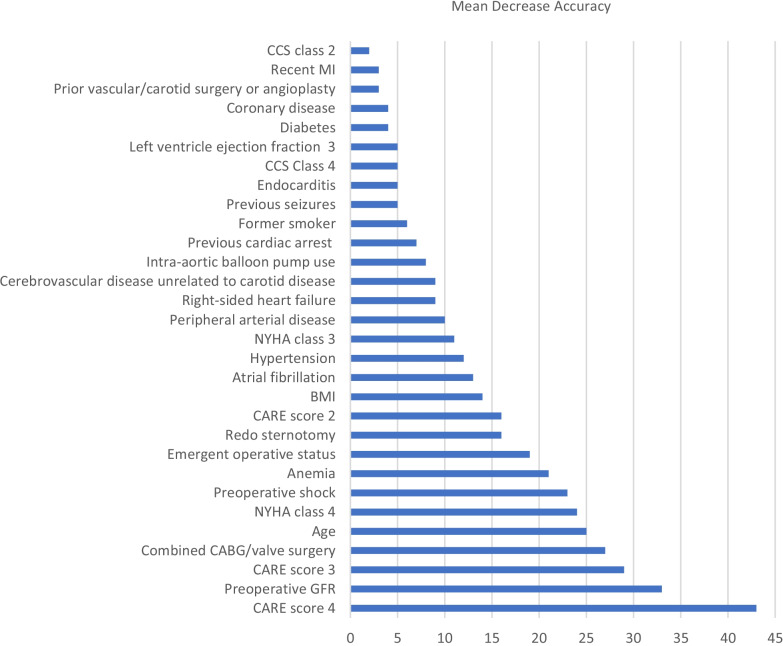
Description of the top 30 variables for prediction of AKI after cardiac surgery. Abbreviations: *CCS class*, Canadian Cardiovascular Society (CCS) grading of angina severity; *Recent MI*, Recent MI within 30 days of surgery; *NYHA class*, New York Heart Association Function class; *BMI*, body mass index; *CARE score*, Cardiac Anesthesia Risk Evaluation (CARE) mortality risk score; *CABG*, Coronary artery bypass grafting; *GFR*, glomerular filtration rate

The accuracy of the Random Forests model was 92.8% in derivation sample, and 75.5% after tenfold cross-validation. The resulting top 30 predictor variables are summarized in Fig. 1.

After applying high-performance logistic regression to achieve parsimony, the final ML model consisted of 12 variables, including: CARE score (2–4), BMI, hypertension, atrial fibrillation, NYHA Class 3, left ventricle ejection fraction < 35%, anemia, emergent operative status, redo sternotomy, combined CABG/valve surgery, former smoker, and preoperative intra-aortic balloon pump use (Table 1).

**Table 1:** The risk model of AKI derived through a hybrid Machine Learning approach

Characteristic	AOR, 95% CI	β coefficient	Risk score
*CARE score—2*			
No	Ref	Ref	0
Yes	1.95 (1.61–2.36)	0.3284	3
*CARE score—3*			
No	Ref	Ref	0
Yes	2.97 (2.25–3.92)	0.5143	5
*CARE score—4*			
No	Ref	Ref	0
Yes	6.43 (1.58–9.70)	0.9298	8
*Combined CABG/valve surgery*			
No	Ref	Ref	0
Yes	1.44 (1.20–1.73)	0.1854	2
*Emergent operative status*			
No	Ref	Ref	0
Yes	1.54 (1.14–1.89)	0.2080	2
*Anemia*			
No	Ref	Ref	0
Yes	1.77 (1.48–1.97)	0.2852	3
*Atrial fibrillation*			
No	Ref	Ref	0
Yes	1.43 (1.17–1.63)	0.1778	2
*NYHA Class 3*			
No	Ref	Ref	0
Yes	1.35 (1.17–1.63)	0.1501	1
*Left ventricle ejection fraction—3*			
No	Ref	Ref	0
Yes	1.39 (1.11–1.73)	0.1644	1
*Redo sternotomy*			
No	Ref	Ref	0
Yes	1.40 (1.09–1.79)	0.1778	2
*Hypertension*			
No	Ref	Ref	0
Yes	1.43 (1.21–1.73)	0.2102	2
*Preoperative intra-aortic balloon pump therapy*			
No	Ref	Ref	0
Yes	2.98 (1.66–4.89)	0.5486	5
*Former smoker*			
No	Ref	Ref	0
Yes	1.25 (1.08–1.44)	0.1107	1
*BMI*	1.04 (1.03–1.06)	0.0350	
≤ 18		− 0.385	− 4
19–24		Ref	0
25–29		0.1925	2
≥ 30		0.6125	6

*CARE score*, Cardiac Anesthesia Risk Evaluation (CARE) mortality risk score; *CABG*, Coronary artery bypass grafting; *NYHA class*, New York Heart Association Classification; *BMI*, body mass index

The model performance in the derivation sample is presented in Table 2.

**Table 2:** Performance of the risk models in the derivation dataset

	Discrimination (AUC)	Calibration (Hosmer–Lemeshow)	Performance
Machine Learning model	0.75	χ^2^ = 7.35, *p* = 0.393	Predicted risk of 3%*: Sensitivity = 67.1% Specificity = 94.1% PPV = 50.2% NPV = 87.6%
Traditional logistic regression model	0.72	χ^2^ = 50.69, *p* < 0.001	Predicted risk of 2%*: Sensitivity = 62.2% Specificity = 65.8% PPV = 40.9% NPV = 82.1%
Enhanced logistic regression model	0.74	χ^2^ = 9.65, *p* = 0.290	Predicted risk of 2%*: Sensitivity = 66.3%; Specificity = 79.1%; PPV = 47.5%; NPV = 84.4%

*Predicted probability threshold with the optimal operating characteristics

*AUC*, area under the Receiver-operating characteristics curve; *PPV*, positive prediction value; *NPV*, negative predictive value

The mean of the total risk score was 10.16 (SD = 5.54) across retained covariates. The total risk score was strongly associated with postoperative AKI (OR = 1.20, 95% 1.18–1.22) in univariate logistic regression. The predicted probability threshold with the optimal operating characteristics (e.g., the square of distance between the point (0, 1) on the upper left hand corner of ROC space and any point on ROC curve) [39], was a predicted risk of 3% (sensitivity, 67.1%; specificity, 94.1%; PPV, 50.2%; NPV, 87.6%). Using a predictive probability of 50% yielded the following results: sensitivity, 31.2%; specificity, 94.4%; PPV, 71.1%; and NPV,78.6%. The risk prediction model remained robust after internal validation (AUC = 0.75; H–L χ^2^ = 5.34, *p* = 0.804) (Additional file 8: Figure S3).

### Traditional statistical model

The final traditional model consisted of six predictor variables: CARE score, HF, anemia, smoking, BMI, and redo sternotomy (Table 3).

**Table 3:** The “traditional” risk model of AKI derived through logistic regression with automated backward variable selection

Characteristics	AORs, 95% CI	β coefficient	Risk score
*CARE score*			
1	Ref	Ref	0
2	2.32 (1.95–2.76)	− 0.3139	− 4
3	3.83 (3.02–4.85)	0.2756	3
4	9.64 (6.77–13.73)	1.2220	14
*NYHA Functional Class*			
0	Ref	Ref	0
1	0.85 (0.66–1.10)	− 0.3184	− 4
2	1.10 (0.91–1.34)	− 0.0781	− 1
3	1.31 (1.08–1.59)	0.0889	1
4	1.76 (1.28–2.43)	0.4427	5
*Anemia*			
No	Ref	Ref	0
Yes	1.60 (1.38–1.86)	0.2124	2
*Redo sternotomy*			
No	Ref	Ref	0
Yes	1.45 (1.13–1.86)	0.2427	3
*Smoking status*			
Never	Ref	Ref	0
Current	1.21 (0.98–1.51)	0.1007	1
Former	1.42 (1.21–1.66)	0.1146	1
*BMI*	1.04 (1.03–1.06)	0.0434	
≤ 18		0.4774	− 5
19–24		Ref	0
25–29		0.2387	3
≥ 30		0.7595	8

*CARE score*, Cardiac Anesthesia Risk Evaluation (CARE) mortality risk score; *NYHA class*, New York Heart Association Classification; *BMI*, body mass index

The mean of the total risk score was 8.67 (SD = 16.86). The total risk score was significantly associated with postoperative AKI (OR = 1.04, 95% 1.03–1.05). The model performance in the derivation sample is presented in Table 2. The predicted probability threshold with the optimal operating characteristics [39], was a predicted risk of 2% (sensitivity, 62.2%; specificity, 65.8%; PPV, 40.9%; and NPV, 82.1%). Using a predictive probability of 50% yielded the following results: sensitivity, 12.9%; specificity, 95.7%; PPV, 56.2%; and NPV,73.6%. In the validation sample, the point score model was modestly discriminative (AUC = 0.70), but poor calibrated (H–L χ^2^ = 20.32, *p* < 0.001) (Additional file 9: Figure S4).

### Enhanced statistical model using bootstrapping methods

The final enhanced model consisted of 10 predictor variables, including: CARE score, hypertension, atrial fibrillation, HF, smoking status, BMI, surgery type, redo sternotomy, and preoperative intra-aortic balloon pump use (Table 4).

**Table 4:** The “enhanced” risk model of AKI derived through logistic regression with backward stepwise variable selection using 500 bootstrap samples

Characteristics	AORs, 95% CI	β coefficient	Risk scores
*Surgery type*			
CABG	Ref	Ref	0
Single Valve	1.02 (0.80–1.28)	− 0.0809	− 2
Combined CABG/valves	1.52 (1.24–1.87)	0.2509	5
*CARE score*			
1	Ref	Ref	0
2	1.94 (1.60–2.34)	− 0.2059	− 4
3	2.83 (2.13–3.75)	0.1737	3
4	5.84 (3.93–8.68)	0.8988	18
*Atrial fibrillation*			
No	Ref	Ref	0
Yes	1.48 (1.23–1.78)	0.1959	4
*NYHA functional class*			
0	Ref	Ref	0
1	0.84 (0.65–1.10)	− 0.2797	− 6
2	1.04 (0.85–1.28)	− 0.0673	− 1
3	1.31 (1.07–2.08)	0.1610	3
4	1.50 (1.08–2.08)	0.2951	6
*Hypertension*			
No	Ref	Ref	0
Yes	1.50 (1.25–1.79)	0.2019	4
*Anemia*			
No	Ref	Ref	0
Yes	1.70 (1.46–1.98)	0.2652	5
*Preoperative intra-aortic balloon pump therapy*			
No	Ref	Ref	0
Yes	3.28 (1.84–5.82)	0.5938	12
*Redo sternotomy*			
No	Ref	Ref	0
Yes	1.45 (1.13–1.87)	0.1857	4
*Smoking status*			
Never	Ref	Ref	0
Current	1.33 (1.07–1.66)	0.1185	2
Former	1.24 (1.06–1.46)	0.0497	1
*BMI*	1.03 (1.01–1.04)	0.0280	
≤ 18		− 0.308	− 6
19 ≤ BMI ≤ 24		Ref	0
25 ≤ BMI ≤ 29		0.154	3
≥ 30		0.490	10

*CARE score*, Cardiac Anesthesia Risk Evaluation (CARE) mortality risk score; *NYHA class*, New York Heart Association Classification; *BMI*, body mass index

The mean of the total risk score was 11.16 (SD = 15.24). The total risk score was significantly associated with AKI (OR = 1.16, 95% 1.14–1.17 The model performance in the derivation sample is presented in Table 2. The predicted probability threshold with the optimal operating characteristics [39], was a predicted risk of 2% (sensitivity, 66.3%; specificity, 79.1%; PPV, 47.5%; and NPV, 84.4%). Using a predictive probability of 50% yielded the following results: sensitivity, 24.3%; specificity, 96.4%; PPV, 66.3%; and NPV, 76.6%. The risk prediction model remained robust after internal validation (AUC = 0.74; H–L χ^2^ = 8.9442, *p* = 0.347) (Additional file 10: Figure S5).

## Discussion

To our knowledge, this study is the first to date that uses a hybrid ML approach to derive and validate a model to predict cardiac surgery-associated AKI of any severity, using only preoperative variables. Our findings suggest that a hybrid ML algorithm predicts better, and is computationally more efficient, than traditional and enhanced techniques for risk modeling.

Previous research has shown that the use of automated variable selection methods could result in the selection of non-reproducible sets of independent variables, thus biasing the estimated regression coefficients [40]. Because of this, the use of backward variable selection in repeated bootstrap samples would likely result in improved estimation of regression coefficients with narrower confidence intervals [30]. Our hybrid ML approach benefits form its ability to accommodate inter-correlation between multiple explanatory variables and providing protection from over-fitting the data [15], and thus, outperforms both traditional and enhanced regression models.

Several cardiac surgery-associated AKI risk models have been proposed to date, with the models predicting renal replacement therapy being most robust [9–11]. Despite the clinical importance of renal replacement therapy, its low incidence rate (2–3%), late occurrence [41], and end stage physiology limit the practical benefit of these risk models. In contrast, mild AKI is very common (pooled incidence rate of 22.3%) [42] and contributes to considerable perioperative and long-term morbidity and mortality [14]. The kidneys are sensitive to unfavorable physiologic processes in the setting of cardiac surgery, which include hypotension, low cardiac output syndrome, systemic inflammation resulting from the mechanical trauma of extracorporeal red blood cell in contact with artificial surfaces [43, 44], as well as the catecholamine surge, decreased vasomotor reactivity and the mismatch of medullary blood flow and renal oxygen consumption that occur during the post-bypass period. Taken together, accurate *preoperative* prediction of AKI of any severity, prior to exposure to intra- and post-operative stresses, affords clinicians the greatest window of opportunity to proactively intensify physiologic monitoring, personalizing fluid management and hemodynamic goals to optimize systemic and renal perfusion in at-risk patients [18].

We used KDIGO to define AKI [19], which enables standardization of reporting and compatibility with similar studies. Our high quality, comprehensive clinical databases provided a large number of standardized candidate variables for ML and statistical modeling. Our ML risk model contains 11 variables that are etiologically associated with AKI after cardiac surgery [12]. We found that our ML model was more accurate than the traditional and enhanced statistical models (AUC = 0.75 vs. 0.70 and 0.73, respectively).

In addition, the ML and enhanced statistical models were well calibrated, while the traditional statistical model was not. From a practical perspective, the ML model was more computationally efficient than the enhanced backward selection algorithm using 500 bootstrap samples. Our findings are consistent with the literature, where recent medical applications of ML have shown a high degree of accuracy in predicting various outcomes across a spectrum of clinical settings and diseases [45, 46].

Few published studies to date predicted cardiac surgery-associated AKI of any severity. Our ML risk model had a higher predictive ability and was more parsimonious (AUC = 0.75, H–L *p* = 0.804) than a recent preoperative model for cardiac surgery-associated AKI of any severity (AUC = 0.73, H–L *p* = 0.490) [20], which was derived using a traditional statistical approach and consisted of 15 risk factors. This model was developed using prospectively collected data from over 30,000 subjects undergoing cardiac surgery at three hospitals in the UK and was externally validated. Our ML model also had similar predictive accuracy and better calibration compared to another contemporary preoperative risk score [22] for any-stage AKI consisted of 10 risk factors (AUC = 0.77, H–L *p* = 0.06), that was derived using bootstrapping methods and was validated internally. It is to be noted that in the latter model, AKI was defined as that occurring within 30 days of cardiac surgery. This definition likely captures events occurring during surgical readmissions or during complicated and prolonged postoperative stays. These events may be unrelated to the index surgery and may thus be impractical for informing preventative therapy in the intraoperative setting.

Two other published risk models for predicting AKI of any severity after cardiac surgery combined various pre-, intra- and postoperative factors [13, 47]. These studies demonstrate that the addition of perioperative factors could improve model performance (AUC = 0.84, and AUC = 0.81, respectively). Further research could be aimed to investigate the additive predictive value of key perioperative variables such as hypotension and low cardiac output, to produce “staged models”. Such models would inform preoperative AKI risk stratification for the planning and personalization of pre- and intraoperative management, as well as to enhance prognostication based on intra- and post-operative events.

Clinical prediction models and associated risk-scoring systems are popular statistical methods as they permit a rapid assessment of patient risk without the use of computers or other electronic devices [48]. The additive point score assigned to each predictor in the developed models to predict AKI of any severity was derived from well-fit logistic regression models, and can readily be applied at the bedside. These validated scores to predict AKI of any severity following cardiac surgery will aid in clinical decision-making, patient counseling and informed decision-making, resource utilization, and preoperative medical optimization [12]. Future research is recommended to prospectively assess the efficacy of these models to enhance personalized fluid and hemodynamic management, as well as minimizing exposure to nephrotoxins, in preventing perioperative AKI.

Our findings should be interpreted in light of several limitations. *First*, our study was conducted in the setting of a single tertiary care hospital. Therefore, our ML model needs to be externally validated before it can confidently be used at other institutions and geographic regions. *Second*, a relatively small number of covariates was included in this study. The performance of the Random Forests approach may be improved in the presence of a larger distribution of covariates [49]. *Third*, our risk model is tailored to patients undergoing procedures involving cardiopulmonary bypass and may not be applicable in the setting of off-pump CABG [50]. *Forth*, we did not incorporate urine output criteria in identifying patients with AKI, because this information was not available in our databases. *Finally*, unmeasured confounding characteristics are an important consideration in any retrospective analysis.

## Conclusions

In summary, we derived and internally validated an accurate and well-calibrated preoperative risk model for cardiac surgery-associated AKI of any severity. We found in this study that risk modeling using a hybrid ML approach led to better model performance than parametric statistical approaches, without sacrifice of computational efficiency. Further studies are needed to externally validate this model, as well as to derive and validate staged models to better inform management and prognostication.

## Supplementary Information

**Additional file 1. Table S1.** Preoperative risk factors.

**Additional file 2. Table S2.** Baseline characteristics in patients with and without postoperative AKI.

**Additional file 3. Table S3.** Baseline characteristics in patients with and without postoperative AKI, in derivation/validation samples.

**Additional file 4. Table S4.** Univariate and multivariate association of predictors with postoperative AKI.

**Additional file 5.** Point score system using the method described by Sullivan et al.

**Additional file 6. Figure S1.** Random Forests algorithm.

**Additional file 7. Figure S2.** Data partitioning in Random Forests.

**Additional file 8. Figure S3.** Receiver-operating characteristic (ROC) curve and calibration plot of the machine learning acute kidney injury risk model, in validation dataset.

**Additional file 9. Figure S4.** Receiver-operating characteristic (ROC) curve and calibration plot of the traditional logistic regression acute kidney injury risk model, in validation dataset.

**Additional file 10. Figure S5.** Receiver-operating characteristic (ROC) curve and calibration plot of the enhanced logistic regression acute kidney injury risk model, in validation dataset.

**Additional file 11.** Review history files.

## Data Availability

The datasets analyzed during the current study are available from University of Ottawa Heart Institute Research Corporation, but restrictions apply to the availability of these data, which were used under license for the current study, and so are not publicly available. Data are however available from the authors upon reasonable request and with permission of University of Ottawa Heart Institute Research Corporation.

## Abbreviations

AKI: Acute kidney injury
ML: Machine Learning
AI: Artificial Intelligence
CABG: Coronary artery bypass graft surgery
KDIGO: Kidney Disease: Improving Global Outcomes
CARE: Cardiac Anesthesia Risk Evaluation
CCS: Canadian Cardiovascular Society
NYHA: New York Heart Association
H–L: Hosmer–Lemeshow
PPV: Positive predictive value
NPV: Negative predictive value
VIM: Variable importance measure
AOR: Adjusted odds ratio
CI: Confidence interval
AUC: Area under curve
ROC curve: Receiver operating characteristic curve
