# Supplementary material for: Prediction of acute kidney injury risk after cardiac surgery: using a hybrid machine learning algorithm
Source: BMC Med Inform Decis Mak. 2022 May 18;22:137. doi: 10.1186/s12911-022-01859-w (PMC9118758; doi:10.1186/s12911-022-01859-w)
Supplement: Supplementary file 1 — Additional file 1. Table S1. Preoperative risk factors. [file 12911_2022_1859_MOESM1_ESM.docx]

**Table S1. Preoperative risk factors**

| **Characteristics** | **Type of variable** |
| --- | --- |
| Acute kidney surgery, n (%) | Categorical – Yes/No |
| Age | Continuous |
| Sex, n (%) | Categorical – Male/Female |
| BMI | Continuous |
| ^a^ Preoperative eGFR | Continuous |
| Surgery type | Categorical:   - CABG - Single Valve - Combined CABG/valve |
| ^b^CARE score, n (%) | Categorical:   1. Stable cardiac disease, no other medical problems/or one or more controlled medical problems, undergoing noncomplex surgery 2. Any uncontrolled medical problem OR undergoing complex surgery 3. Any uncontrolled medical problem AND undergoing complex surgery 4. Chronic or advanced cardiac disease undergoing cardiac surgery as a last hope to save or improve life |
| Emergent operative status, n (%) | Categorical: emergent/elective |
| CCS grading of angina severity, n (%) | Categorical:  0 None   1. Angina with strenuous or rapid or prolonged exertion at work or recreation 2. Slight limitation of ordinary activity 3. Marked limitation of ordinary physical activity 4. Inability to carry on any physical activity without discomfort, anginal syndrome may be present at rest |
| Atrial fibrillation, n (%) | Categorical – Yes/No |
| New York Heart Association functional class, n (%) | Categorical:   1. No 2. No limitation of physical activity. Ordinary physical activity does not cause undue fatigue, palpitation, dyspnea 3. Slight limitation of physical activity. Comfortable at rest. Ordinary physical activity results in fatigue, palpitation, dyspnea 4. Marked limitation of physical activity. Comfortable at rest. Less than ordinary activity causes fatigue, palpitation, or dyspnea 5. Unable to carry on any physical activity without discomfort. Symptoms of heart failure at rest. If any physical activity is undertaken, discomfort increases |
| Recent MI within 30 days of surgery, n (%) | Categorical – Yes/No |
| Left ventricular ejection fraction class, n (%) | Categorical:   1. ≥50% 2. 35-50% 3. 20-34% 4. <20% |
| Hypertension, n (%) | Categorical – Yes/No |
| Prior vascular/carotid surgery or angioplasty, n (%) | Categorical – Yes/No |
| Cerebrovascular disease unrelated to carotid disease, n (%) | Categorical – Yes/No |
| Cerebrovascular disease related to carotid disease, n (%) | Categorical – Yes/No |
| Diabetes, n (%) | Categorical – Yes/No |
| Carotid disease, n (%) | Categorical – Yes/No |
| Peripheral arterial disease, n (%) | Categorical – Yes/No |
| Coronary artery disease, n (%) | Categorical – Yes/No |
| Presence of residual neurologic deficit after stroke, n (%) | Categorical – Yes/No |
| Anemia, n (%) | Categorical – Yes/No |
| Preoperative cardiogenic shock, n (%) | Categorical – Yes/No |
| Previous cardiac arrest, n (%) | Categorical – Yes/No |
| Preoperative Intra-aortic balloon pump therapy, n (%) | Categorical – Yes/No |
| Right-sided heart failure, n (%) | Categorical – Yes/No |
| Redo sternotomy, n (%) | Categorical – Yes/No |
| Infective endocarditis, n (%) | Categorical – Yes/No |
| Seizure disorder, n (%) | Categorical – Yes/No |
| Smoking status, n (%) | Categorical:   - Never - Current - Former |
| Alcoholism, n (%) | Categorical:   - Never - Current - Former |

^a^Preoperative glomerular filtration rate was calculated using The Chronic Kidney Disease Epidemiology Collaboration creatinine equation

^b^The Cardiac Anesthesia Risk Evaluation (CARE) score that is a validated mortality score that integrated the severity of cardiac disease, co-existing medical problems and complexity of cardiac surgery to be performed (1)

BMI=body mass index

CCS= Canadian Cardiovascular Society
