# Supplementary material for: Prediction of acute kidney injury risk after cardiac surgery: using a hybrid machine learning algorithm
Source: BMC Med Inform Decis Mak. 2022 May 18;22:137. doi: 10.1186/s12911-022-01859-w (PMC9118758; doi:10.1186/s12911-022-01859-w)
Supplement: Supplementary file 2 — Additional file 2. Table S2. Baseline characteristics in patients with and without postoperative AKI. [file 12911_2022_1859_MOESM2_ESM.docx]

**Figure S1. Random Forests algorithm**

**
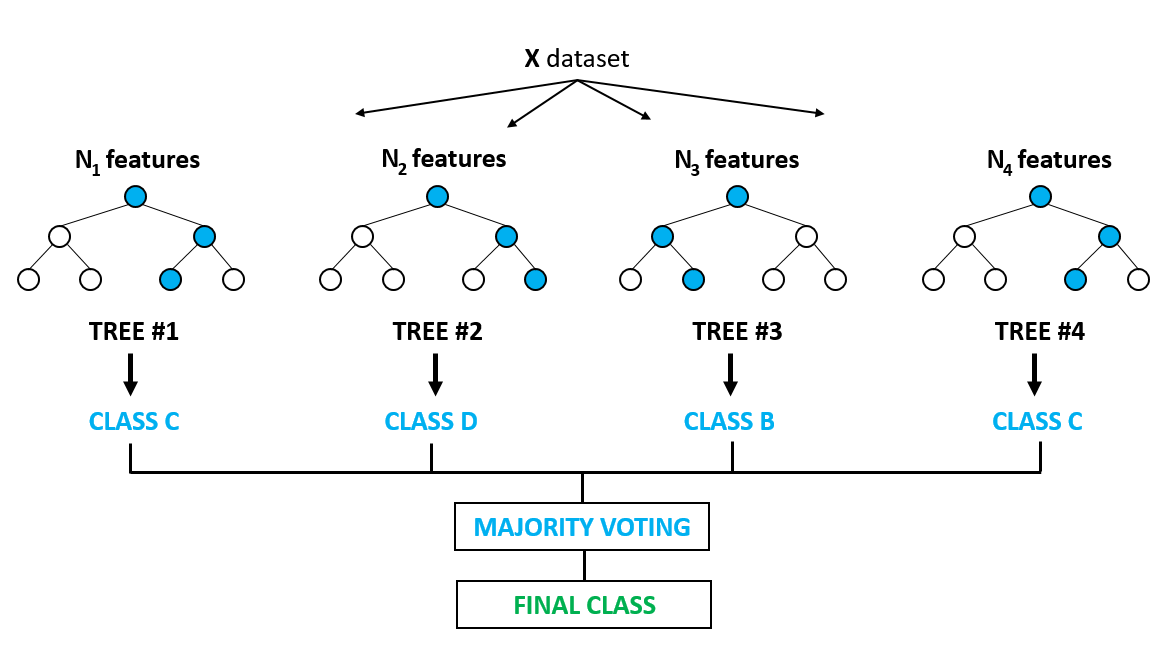
**

Bootstrapping is an uncertainty modelling step that generates modified versions of the training set over which trees will be grown and ensemble predictions averaged. Trees are grown using binary portioning. Once a node is split on the best eligible splitter, the process is repeated in its entirety on each child node. A new list of eligible predictors is selected at random for each node. When multiple models are generated, they are normally combined by ‘voting’. For every record, the proportion of votes for each class (variable) represents the probability of class membership. Winner is the class with the most votes.
