# Supplementary material for: Prediction of acute kidney injury risk after cardiac surgery: using a hybrid machine learning algorithm
Source: BMC Med Inform Decis Mak. 2022 May 18;22:137. doi: 10.1186/s12911-022-01859-w (PMC9118758; doi:10.1186/s12911-022-01859-w)
Supplement: Supplementary file 3 — Additional file 3. Table S3. Baseline characteristics in patients with and without postoperative AKI, in derivation/validation samples. [file 12911_2022_1859_MOESM3_ESM.docx]

**Figure S2. Data partitioning in Random Forests**

**Original dataset**

n=**6,522**

**Validation Partition**

n=1.956

**Derivation Partition**

n=4.566

**Out of Bag**

(1/3 of derivation set)

**Train data**

(2/3 of derivation) **set)**
