# Supplementary material for: Prediction of acute kidney injury risk after cardiac surgery: using a hybrid machine learning algorithm
Source: BMC Med Inform Decis Mak. 2022 May 18;22:137. doi: 10.1186/s12911-022-01859-w (PMC9118758; doi:10.1186/s12911-022-01859-w)
Supplement: Supplementary file 4 — Additional file 4. Table S4. Univariate and multivariate association of predictors with postoperative AKI. [file 12911_2022_1859_MOESM4_ESM.docx]

**Additional file 4. Point score system using the method described by Sullivan et al.**

First, we break the only continues variable, BMI, of the final model into 4 groups: underweight (BMI<=18), normal (BMI from 19 to 24), overweight (BMI from 25 to 29) and obese (BMI more or equal to 30). The group with normal BMI was used as the base category. Reference values for the other three groups were defined as the differences between their mid-point values and 21.5. Then the distance of each age group from the base age category in regression coefficient units was computed by multiplying its reference value by the regression coefficient of BMI. All other risk factors in the model were categorical variables, and the distance between a variable and its base category in regression coefficient units was equal to the size of the coefficient. For each risk factor, its distance from the base category in regression coefficient units was divided by this constant and rounded to the nearest integer to get its point value.
