# Supplementary material for: Prediction of acute kidney injury risk after cardiac surgery: using a hybrid machine learning algorithm
Source: BMC Med Inform Decis Mak. 2022 May 18;22:137. doi: 10.1186/s12911-022-01859-w (PMC9118758; doi:10.1186/s12911-022-01859-w)
Supplement: Supplementary file 5 — Additional file 5. Point score system using the method described by Sullivan et al. [file 12911_2022_1859_MOESM5_ESM.docx]

**Table S2. Baseline characteristics in patients with and without postoperative AKI.**

| **Characteristics** | **Overall cohort**  **n=6,522** | **AKI**  **n=1,760** | **Non AKI**  **n=4,762** |
| --- | --- | --- | --- |
| Age, mean (SD) | 65.8 (11.8) | 67.1 (11.9) | 65.4 (11.7) |
| Male, n (%) | 4,698 (72.0) | 1,262 (71.7) | 3,436 (72.1) |
| BMI, mean (SD) | 28.5 (4.3) | 28.8 (4.7) | 28.3 (4.2) |
| Estimated GFR, mean (SD) | 87.1 (37.0) | 80.4 (38.4) | 89.5 (36.1) |
| Surgery type |  |  |  |
| Single valve | 2,863 (43.9) | 526 (29.9) | 2,337 (49.1) |
| Valves | 1.169 (17.9) | 243 (13.8) | 926 (19.5) |
| Valve/CABG (combined) | 2,490 (38.2) | 991 (56.3) | 1,499 (31.5) |
| CARE score, n (%) |  |  |  |
| 1 | 2,750 (42.2) | 351 (20.0) | 2,399 (50.5) |
| 2 | 2,567 (39.4) | 758 (43.1) | 1,809 (38.0) |
| 3 | 864 (13.3) | 422 (24.0) | 442 (9.3) |
| 4 | 332 (5.1) | 227 (12.9) | 105 (2.2) |
| Emergent operative status, n (%) | 514 (7.9) | 273 (15.5) | 241 (5.1) |
| CCS class, n (%) |  |  |  |
| 0 | 2,697 (41.4) | 810 (46.1) | 1,887 (39.7) |
| 1 | 485 (7.5) | 108 (6.2) | 377 (7.9) |
| 2 | 1,061 (16.3) | 228 (13.0) | 833 (17.5) |
| 3 | 1,086 (16.7) | 263 (15.0) | 823 (17.3) |
| 4 | 1,181 (18.1) | 348 (20.0) | 833 (17.5) |
| Atrial fibrillation, n (%) | 1,091 (16.7) | 453 (74.3) | 638 (25.7) |
| NYHA class, n (%) |  |  |  |
| 0 | 2,476 (38.0) | 472 (26.8) | 2,004 (42.1) |
| 1 | 760 (11.6) | 132 (7.5) | 628 (13.2) |
| 2 | 1,417 (21.7) | 367 (20.9) | 1,050 (22.1) |
| 3 | 1,494 (22.9) | 572 (32.5) | 922 (19.4) |
| 4 | 375 (5.8) | 217 (12.3) | 158 (3.3) |
| Recent MI within 30 days of surgery, n (%) | 1,371 (21.0) | 386 (21.9) | 985 (20.7) |
| Left ventricular ejection fraction, n (%) |  |  |  |
| ≥50% | 4,920 (75.4) | 1,181 (67.1) | 3,739 (78.5) |
| 35-50% | 982 (15.1) | 297 (16.9) | 685 (14.4) |
| <34% | 620 (9.5) | 282 (16.0) | 338 (7.1) |
| History of hypertension, n (%) |  |  |  |
| Yes | 4,766 (73.1) | 1,346 (76.5) | 3,420 (71.8) |
| Prior vascular/carotid surgery or angioplasty, n (%) |  |  |  |
| Yes | 314 (4.8) | 104 (5.9) | 210 (4.4) |
| Cerebrovascular disease unrelated to carotid disease, n (%) |  |  |  |
| Yes | 492 (7.5) | 184 (10.5) | 308 (6.5) |
| Cerebrovascular disease related to carotid disease, n (%) |  |  |  |
| Yes | 236 (3.6) | 81 (4.6) | 155 (3.3) |
| Diabetes, n (%) |  |  |  |
| Yes | 2,016 (30.9) | 596 (33.9) | 1,420 (29.8) |
| Carotid disease, n (%) |  |  |  |
| Yes | 527 (8.1) | 184 (10.4) | 343 (7.2) |
| Peripheral arterial disease, n (%) |  |  |  |
| Yes | 696 (10.7) | 240 (13.6) | 456 (9.6) |
| Coronary artery disease, n (%) |  |  |  |
| Yes | 4,671 (71.6) | 1,244 (70.7) | 3,427 (72.0) |
| Residual neurologic deficit after stroke, n (%) |  |  |  |
| Yes | 135 (2.1) | 56 (3.2) | 79 (1.7) |
| Anemia, n (%) |  |  |  |
| Yes | 2,150 (33.0) | 843 (47.9) | 1,307 (27.5) |
| Preoperative cardiogenic shock, n (%) |  |  |  |
| Yes | 223 (3.4) | 144 (8.2) | 79 (1.7) |
| Previous cardiac arrest, n (%) |  |  |  |
| Yes | 144 (2.2) | 69 (3.9) | 75 (1.6) |
| Intra-aortic balloon pump therapy, n (%) |  |  |  |
| Yes | 90 (1.4) | 60 (3.4) | 30 (0.6) |
| Right-sided heart failure, n (%) |  |  |  |
| Yes | 212 (3.3) | 119 (6.8) | 93 (2.0) |
| Redo sternotomy, n (%) |  |  |  |
| Yes | 525 (8.1) | 260 (14.8) | 265 (5.6) |
| Preoperative endocarditis, n (%) |  |  |  |
| Yes | 115 (1.8) | 62 (3.5) | 53 (1.1) |
| Previous seizures, n (%) |  |  |  |
| Yes | 79 (1.2) | 25 (1.4) | 54 (1.1) |
| Smoking status, n (%) |  |  |  |
| Never | 2,407 (36.9) | 582 (38.3) | 1,825 (38.3) |
| Current | 1,054 (16.2) | 276 (16.3) | 778 (16.3) |
| Former | 3,061 (46.9) | 902 (51.3) | 2,159 (45.3) |
| Alcoholism status, n (%) |  |  |  |
| Never | 6,204 (95.1) | 1,659 (94.3) | 4,545 (95.5) |
| Current | 141 (2.2) | 49 (2.8) | 92 (1.9) |
| Former | 176 (2.7) | 52 (2.9) | 124 (2.6) |

Abbreviations: BMI=body mass index, GFR=glomerular filtration rate, CABG, CARE score, CCS= Canadian Cardiovascular Society, NYHA class= New York Heart Association Function Class, MI=myocardial infarction
