# Supplementary material for: Prediction of acute kidney injury risk after cardiac surgery: using a hybrid machine learning algorithm
Source: BMC Med Inform Decis Mak. 2022 May 18;22:137. doi: 10.1186/s12911-022-01859-w (PMC9118758; doi:10.1186/s12911-022-01859-w)
Supplement: Supplementary file 6 — Additional file 6. Figure S1. Random Forests algorithm. [file 12911_2022_1859_MOESM6_ESM.docx]

**Table S3. Baseline characteristics in patients with and without postoperative AKI, in derivation/validation samples**

| **Characteristics** | **Overall cohort**  **n=6,522** | **Derivation**  **n=4,566** | **Validation**  **n=1,956** |
| --- | --- | --- | --- |
| Acute kidney surgery, n (%) |  |  |  |
| Yes | 1,760 (27.0) | 1,222 (26.7) | 538 (27.5) |
| Age |  |  |  |
| Mean (SD) | 65.8 (11.8) | 65.7 (11.8) | 66.2 (11.7) |
| Sex, n (%) |  |  |  |
| Male | 4,698 (72.0) | 3,295 (72.2) | 1,403 (71.7) |
| BMI |  |  |  |
| Mean (SD) | 28.5 (4.3) | 28.4 (4.3) | 28.5 (4.4) |
| Preop. eGFR |  |  |  |
| Mean (SD) | 87.1 (37.0) | 87.4 (37.2) | 86.3 (36.3) |
| Surgery type |  |  |  |
| Valve/CABG (combined) | 2,863 (43.9) | 1,999 (43.8) | 864 (44.2) |
| Single valve | 1.169 (17.9) | 812 (17.8) | 357 (18.2) |
| Valves | 2,490 (38.2) | 1,755 (38.4) | 735 (37.6) |
| CARE score, n (%) |  |  |  |
| 0-1 | 2,750 (42.2) | 1,937 (42.5) | 813 (41.6) |
| 2 | 2,567 (39.4) | 1,779 (39.0) | 788 (40.3) |
| 3 | 864 (13.3) | 603 (13.2) | 261 (13.4) |
| 4 | 332 (5.1) | 241 (5.3) | 91 (4.7) |
| Urgency of surgery, n (%) |  |  |  |
| Emergent | 514 (7.9) | 356 (7.8) | 158 (8.1) |
| Angina class, n (%) |  |  |  |
| 0 | 2,697 (41.4) | 1,903 (41.8) | 794 (40.7) |
| 1 | 485 (7.5) | 321 (7.0) | 164 (8.4) |
| 2 | 1,061 (16.3) | 734 (16.1) | 327 (16.7) |
| 3 | 1,086 (16.7) | 790 (17.3) | 296 (15.2) |
| 4 | 1,181 (18.1) | 809 (17.8) | 372 (19.0) |
| Arterial fibrillation, n (%) |  |  |  |
| Yes | 1,091 (16.7) | 745 (16.3) | 346 (17.7) |
| Congestive Heart Failure, n (%) |  |  |  |
| No | 2,476 (38.0) | 1,733 (38.0) | 743 (38.0) |
| NYHA I | 760 (11.6) | 532 (11.6) | 228 (11.7) |
| NYHA II | 1,417 (21.7) | 1,000 (21.9) | 417 (21.3) |
| NYHA III | 1,494 (22.9) | 1,041 (22.8) | 453 (23.2) |
| NYHA IV | 375 (5.8) | 260 (5.7) | 115 (5.9) |
| Recent MI within 30 days of surgery, n (%) |  |  |  |
| Yes | 1,371 (21.0) | 926 (20.3) | 445 (22.7) |
| Ventricle ejection fraction, n (%) |  |  |  |
| ≥52 | 4,920 (75.4) | 3,445 (75.5) | 1,475 (75.4) |
| 41-51 | 982 (15.1) | 681 (14.9) | 301 (15.4) |
| <40 | 620 (9.5) | 440 (9.6) | 180 (9.2) |
| Hypertension, n (%) |  |  |  |
| Yes | 4,766 (73.1) | 3,314 (72.6) | 1,452 ()74.2 |
| Prior vascular/carotid surgery or angioplasty, n (%) |  |  |  |
| Yes | 314 (4.8) | 230 (5.0) | 84 (4.3) |
| CVA/TIA unrelated to carotid disease, n (%) |  |  |  |
| Yes | 492 (7.5) | 334 (7.3) | 158 (8.1) |
| CVA/TIA related to carotid disease, n (%) |  |  |  |
| Yes | 236 (3.6) | 162 (3.5) | 74 (3.7) |
| Diabetes, n (%) |  |  |  |
| Yes | 2,016 (30.9) | 1.417 (31.0) | 599 (30.6) |
| Peripheral vascular disease, n (%) |  |  |  |
| Yes | 696 (10.7) | 481 (10.5) | 215 (11.0) |
| Coronary disease, n (%) |  |  |  |
| Yes | 4,671 (71.6) | 3.258 (71.4) | 1 413 (72.2) |
| Post-CVA residual deficit, n (%) |  |  |  |
| Yes | 135 (2.1) | 89 (2.0) | 46 (2.3) |
| Anemia, n (%) |  |  |  |
| Yes | 2,150 (33.0) | 1,519 (33.3) | 631 (32.3) |
| Shock, n (%) |  |  |  |
| Yes | 223 (3.4) | 149 (3.3) | 74 (3.7) |
| Previous cardiac arrest, n (%) |  |  |  |
| Yes | 144 (2.2) | 102 (2.2) | 42 (2.1) |
| Intra-aortic balloon pump therapy, n (%) |  |  |  |
| Yes | 90 (1.4) | 63 (1.4) | 27 (1.4) |
| Right heart failure, n (%) |  |  |  |
| Yes | 212 (3.3) | 155 (3.4) | 57 (2.9) |
| Redo sternotomy, n (%) |  |  |  |
| Yes | 525 (8.1) | 373 (8.2) | 152 (7.8) |
| Preoperative endocarditis, n (%) |  |  |  |
| Yes | 115 (1.8) | 83 (1.8) | 32 (1.6) |
| Previous seizures, n (%) |  |  |  |
| Yes | 79 (1.2) | 63 (1.4) | 16 (0.8) |
| Smoking status, n (%) |  |  |  |
| Never | 2,407 (36.9) | 1,676 (36.7) | 731 (37.4) |
| Current | 1,054 (16.2) | 755 (16.5) | 299 (15.3) |
| Former | 3,061 (46.9) | 2,135 (47.7) | 926 (47.3) |
| Alcoholism status, n (%) |  |  |  |
| Never | 6,204 (95.1) | 4,342 (95.1) | 1,862 (95.2) |
| Current | 141 (2.2) | 104 (2.3) | 37 (1.9) |
| Former | 176 (2.7) | 119 (2.6) | 57 (2.9) |

Abbreviations: BMI=body mass index, GFR=glomerular filtration rate, CABG, CARE score, CCS= Canadian Cardiovascular Society, NYHA class= New York Heart Association Function Class, MI=myocardial infarction
