# Supplementary material for: Prediction of acute kidney injury risk after cardiac surgery: using a hybrid machine learning algorithm
Source: BMC Med Inform Decis Mak. 2022 May 18;22:137. doi: 10.1186/s12911-022-01859-w (PMC9118758; doi:10.1186/s12911-022-01859-w)
Supplement: Supplementary file 7 — Additional file 7. Figure S2. Data partitioning in Random Forests. [file 12911_2022_1859_MOESM7_ESM.docx]

**Table S4. Univariate and multivariate association of predictors with postoperative AKI**

| **Characteristics** | **Unadjusted ORs,**  **95% CI**  n=6,522 | **Adjusted ORs,**  **95% CI**  n=6,522 |
| --- | --- | --- |
| Age | 1.01 (1.00-1.02) | 1.00 (0.99-1.01) |
| Male | 0.98 (0.87-1.10) | 1.2 (1.01-1.34) |
| BMI | 1.02 (1.01-1.04) | 1.04 (1.03-1.06) |
| Preoperative eGFR | 0.99 (0.98-1.00) | 0.99 (0.98-1.01) |
| Surgery type |  |  |
| Valves | 1.16 (0.98-1.38) | 1.18 (0.93-1.50) |
| Combined CABG/valve surgery | 2.91 (2.57-3.30) | 1.58 (1.29-1.93) |
| CARE score |  |  |
| 2 | 2.89 (2.51-3.32) | 1.90 (160-2.25) |
| 3 | 6.51 (5.47-7.76) | 2.62 (2.03-3.37) |
| 4 | 13.94 (10.74-18.09) | 5.11 (3.48-7.50) |
| Emergent operative status | 3.30 (2.73-3.98) | 1.16 (1.01-1.34) |
| CCS class |  |  |
| 1 | 0.68 (0.54-0.86) | 1.01 (0.78- 1.31) |
| 2 | 0.65 (0.55-0.77) | 1.14 (0.93-1.40) |
| 3 | 0.76 (0.64-0.89) | 1.22 (0.99-1.49) |
| 4 | 0.98 (0.84-1.14) | 1.08 (0.86-1.36) |
| Atrial fibrillation | 2.28 (1.99-2.61) | 1.43 (1.22-1.73) |
| NYHA class |  |  |
| 1 | 0.89 (0.72-1.10) | 0.78 (0.62-0.98) |
| 2 | 1.52 (1.30-1.78) | 1.17 (0.98-1.39) |
| 3 | 2.69 (1.32-3.11) | 1.39 (1.16-1.66) |
| 4 | 5.84 (4.1-7.33) | 1.35 (0.9-1.84) |
| Recent MI within 30 days of surgery | 1.07 (0.94-1.23) | 1.01 (0.84-1.21) |
| Left ventricular ejection fraction |  |  |
| 35-50% | 1.39 (1.20-1.62) | 1.09 (0.92-2.30) |
| <34% | 3.75 (2.72-5.19) | 1.37 (1.09-1.73) |
| History of hypertension | 1.29 (1.13-1.46) | 1.40 (1.19-1.65) |
| Prior vascular/carotid surgery or angioplasty | 1.34 (1.05-1.71) | 0.83 (0.60-1.15) |
| Cerebrovascular disease unrelated to carotid disease | 1.68 (1.39-2.04) | 1.25 (0.99-1.58) |
| Cerebrovascular disease related to carotid disease | 1.42 (1.08-1.87) | 0.94 (0.63-0.42) |
| Diabetes | 1.22 (1.08-1.37) | 1.00 (0.87-1.16) |
| Carotid disease | 1.51 (1.25-1.82) | 1.39 (1.04-1.82) |
| Peripheral arterial disease | 1.48 (1.25-1.75) | 1.17 (0.93-1.47) |
| Coronary artery disease | 0.96 (0.85-1.08) | 1.02 (0.84-1.23) |
| Residual neurologic deficit after stroke | 1.99 (1.41-2.82) | 1.03 (0.67-1.58) |
| Anemia | 2.43 (2.17-2.73) | 1.53 (1.33-1.75) |
| Preoperative cardiogenic shock | 5.11 (3.83-6.83) | 1.22 (0.78-1.93) |
| Previous cardiac arrest | 2.50 (1.79-3.49) | 1.00 (0.66-1.49) |
| Intra-aortic balloon pump therapy | 5.71 (3.59-9.08) | 2.16 (1.13-4.12) |
| Right-sided heart failure | 3.68 (1.79-4.87) | 0.99 (0.66-1.40) |
| Redo sternotomy | 2.91 (2.42-3.49) | 1.61 (1.30-1.99) |
| Preoperative endocarditis | 3.34 (2.30-4.85) | 1.80 (1.17-2.77) |
| Previous seizures | 1.22 (0.75-1.97) | 1.14 (0.67-1.92) |
| Smoking status |  |  |
| Current | 1.10 (0.93-1.30) | 1.26 (1.04-1.54) |
| Former | 1.31 (1.16-1.49) | 1.27 (1.11-1.46) |
| Alcoholism status |  |  |
| Current | 1.49 (1.05-2.11) | 1.25 (0.83-1.87) |
| Former | 1.09 (0.78-1.53) | 0.79 (0.54-1.16) |

Abbreviations: BMI=body mass index, GFR=glomerular filtration rate, CABG, CARE score, CCS= Canadian Cardiovascular Society, NYHA class= New York Heart Association Function Class, MI=myocardial infarction
