# Supplementary material for: Prediction of acute kidney injury risk after cardiac surgery: using a hybrid machine learning algorithm
Source: BMC Med Inform Decis Mak. 2022 May 18;22:137. doi: 10.1186/s12911-022-01859-w (PMC9118758; doi:10.1186/s12911-022-01859-w)
Supplement: Supplementary file 8 — Additional file 8. Figure S3. Receiver-operating characteristic (ROC) curve and calibration plot of the machine learning acute kidney injury risk model, in validation dataset. [file 12911_2022_1859_MOESM8_ESM.docx]

**Figure S3. Receiver-operating characteristic (ROC) curve and calibration plot of the machine learning acute kidney injury risk model, in validation dataset**


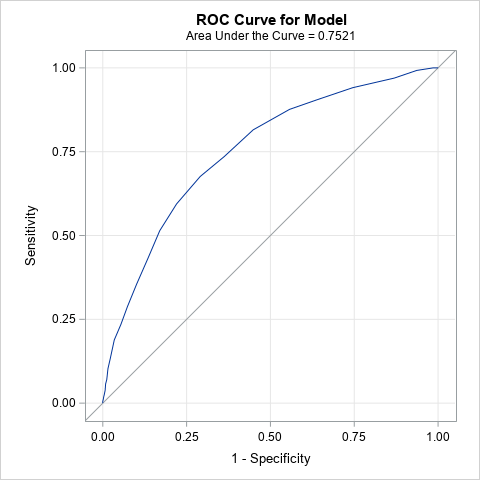

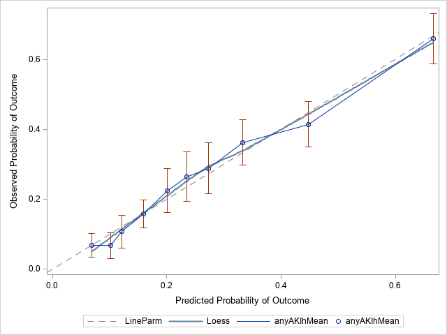


Decile-decile calibration plots of the predicted probability (deciles) versus the observed probability of AKI.
