# Supplementary material for: Prediction of acute kidney injury risk after cardiac surgery: using a hybrid machine learning algorithm
Source: BMC Med Inform Decis Mak. 2022 May 18;22:137. doi: 10.1186/s12911-022-01859-w (PMC9118758; doi:10.1186/s12911-022-01859-w)
Supplement: Supplementary file 11 — Additional file 11. Review history files. [file 12911_2022_1859_MOESM11_ESM.docx]

Version 1 Submitted on 28-Sep-21

# Katja M. Gist - 4-NOV-21

FEEDBACK FOR AUTHOR(S)

Prediction of Acute Kidney Injury Risk After Cardiac Surgery: Using a Hybrid Machine Learning Algorithm

Petrosyan and colleagues performed a retrospective study of adults undergoing cardiac surgery with cardiopulmonary bypass to evaluate 3 risk models: 1) a hybrid machine learning algorithm, traditional logistic regression and an enhanced logistic regression model with 500 bootstraps with backward variable selection for prediction of serum creatinine defined AKI within 7 days of cardiac surgery. The population of 6500 patients was defined into a derivation cohort (2/3 of patients) and validation cohort (1/3 patients). The investigators found that the hybrid machine learning model performed best, and importantly could be used at the bedside.

This is an interesting paper and provides a novel risk stratification tool. The paper is well written and focused, and a preoperative risk stratification tool is probably what is needed to identify different risk profiles for patients who may develop AKI in the first 7 postoperative days. Major and minor comments are discussed below.

Major:

-	The authors only use the serum creatinine criteria for defining AKI. I believe this to be a major limitation. Recent work by Kellum et al, and other existing publications identify that there are a significant proportion of missed cases of AKI by only including creatinine criteria. This is especially important because of the association with clinically relevant outcomes. The authors should consider incorporation of the urine output criteria. There should otherwise be significant justification on why the urine output criteria could not be included.

o	Kellum JA, Sileanu FE, Murugan R, Lucko N, Shaw AD, Clermont G. Classifying AKI by Urine Output versus Serum Creatinine Level. J Am Soc Nephrol. 2015 Sep;26(9):2231-8. doi: 10.1681/ASN.2014070724. Epub 2015 Jan 7. PMID: 25568178; PMCID: PMC4552117.

-	The primary study outcome is stage 1 AKI. While stage 1 AKI is an important outcome, the stronger associations of AKI with outcomes is stage 2 and 3. It is not clear if there is a specific assessment of each of the models with stage 2 or 3 AKI. Furthermore, recommendations by the ADQI 16 suggest that AKI should be classified by both Urine output and creatinine (see #1 above) but also as transient vs persistent. In this manuscript, the authors do not delineate the transient vs. persistent nature of AKI, and certainly how this prediction tool would perform with high severity of AKI, and for the transient vs. persistent disease. The ADQI16 paper reference is below.

o	(Chawla LS, Bellomo R, Bihorac A, Goldstein SL, Siew ED, Bagshaw SM, Bittleman D, Cruz D, Endre Z, Fitzgerald RL, Forni L, Kane-Gill SL, Hoste E, Koyner J, Liu KD, Macedo E, Mehta R, Murray P, Nadim M, Ostermann M, Palevsky PM, Pannu N, Rosner M, Wald R, Zarbock A, Ronco C, Kellum JA; Acute Disease Quality Initiative Workgroup 16. Acute kidney disease and renal recovery: consensus report of the Acute Disease Quality Initiative (ADQI) 16 Workgroup. Nat Rev Nephrol. 2017 Apr;13(4):241-257. doi: 10.1038/nrneph.2017.2. Epub 2017 Feb 27. PMID: 28239173).

-	The authors state that the model could then be used to intensify physiologic monitoring, personalize fluid management and hemodynamic goals to optimize systemic renal perfusion, but it is not clear from multicenter trials if these interventions work. This should be expanded in the discussion, in addition to the contribution of other factors in the postoperative period that exacerbate AKI, like nephrotoxins.

-	The authors state that this “score” an be easily calculated at the bedside. I believe this warrants additional discussion, especially with how the electronic health record can be leveraged for clinical decision support as has been the case for other trials (Persevere) and in pediatrics – Taking focus 2 (https://clinicaltrials.gov/ct2/show/NCT03541785)

Minor:

-	There is a typo of page 8, “goodness-of-fir” on line 3, should be changed to “goodness-of-fit”

# Changwung Jo - 2-DEC-21

FEEDBACK FOR AUTHOR(S)

It was great honor to be asked to review this manuscript for BMC. As with all research endeavors a great amount of work and effort goes into completing a manuscript and it is appreciated. I have included specific comments as well as suggestions.

The Summary:

This is a retrospective cohort study of hybrid ML model that predicts postoperative AKI of adult patients who underwent major cardiac surgery using only preoperative information. The authors developed three AKI risk models and found that the hybrid ML showed the highest AUC among the models. It was an interesting subject, however, there are some problems. Major revisions are needed.

Comments:

First, a comment on the research method.

It seems that only internal validation was performed in this study. External validation was not conducted with data from other organizations, so the universality of the model could not be secured. In addition, the possibility that the model is overfitted cannot be ruled out.

Since external validation was not carried out in this study, it would be good if the model could be provided in a form that could be used by other institutions even for later validation. For example, the model can be published on github, or it can be provided as a web service so that other organizations can use the model. In this way, the universality of the model not confirmed in this study can be secured by sharing the results of other institutions' verification of the model, and it will be able to contribute to academic development.

It was described that the backward method was used for feature selection. What was the result of forward feature selection? What is the difference between the two results? I would like to suggest the reason for adopting backward feature selection should be described in this study.

I wonder if collinearity between variables was analyzed. At least when I read the manuscript, I couldn't find out that variables with high collinearity were excluded. It is hoped that data related to collinearity between variables used in this study will be provided as a supplementary.

Also, there was no mention of how to deal with missing values. I wonder if there were any missing values in the tabular data used in the study. If there is, it would be good to describe how much of the missing value was for each variable in the entire data and how the imputation for the missing value was handled.

Although the above-mentioned contents are very basic contents in ML study, they are contents that must be described in the method to secure the reproducibility of the study.

The next thing is about ML.

First of all, there are various ML models, and I wonder if there was a reason to use random forest among them. If you explain what AUC was like when other models were used, it might be the answer.

Unlike the existing linear model, ML has the advantage that the weight of each variable is flexibly changed according to the input value. Although the random forest algorithm was used, in the end, logistic regression was added after the ramdom forest so that the weights for each variable were always consistent. It is said that it was done to solve the black box problem, but it seems that the harm is greater than the benefit. Rather, it is more efficient to use a random forest model and use eli5, etc. to visualize which variables contributed to the predicted values and how they contributed to the input values, and it would also prevent information loss.

Twelve variables are too many variables to use in general. If you plan to use this model in clinical practice, not just for academic research, you should be able to predict well with fewer variables than this. Even if AUC is somewhat compromised, it seems possible to consider a method to reduce the number of features.

Finally, the difference in AUC between the models proposed in this study seems to be insignificant. AUC 0.74 can already be achieved with the logistic model alone. There is almost no difference in AUC, but it requires more computing power and I doubt whether ML should be used here in many ways. There are data that are suitable for ML and there are data that are not. At least, judging by the given data, it seems likely that this data is not suitable for ML. If it is necessary to use ML, it would be logical to accurately describe the reason for doing so and the differentiated advantages that can be obtained by using ML in this study.

# Editor: Piero Lo Monaco - Revision requested | 04-Jan-22

I am pleased to inform you that your manuscript is potentially acceptable for publication, once you have carried out some essential revisions suggested by our reviewers. Their reports are below.

Please accompany your revised manuscript with a point-by-point response letter providing a detailed response to each reviewer/editorial point raised, describing what amendments have been made to the manuscript text and where these can be found (e.g. Methods section, line 12, page 5). If you disagree with any comments raised, please provide a detailed rebuttal to help explain and justify your decision.

Version 2 Submitted on 20-Feb-22

# Katja M. Gist - 15-MAR-22

FEEDBACK FOR AUTHOR(S)

The authors have addressed all my comments and made changes in the manuscript accordingly.

# Changwung Jo - 22-MAR-22

FEEDBACK FOR AUTHOR(S)

It was great honor to be asked to review this manuscript for BMC. As with all research

endeavors a great amount of work and effort goes into completing a manuscript and it is

appreciated.

The Summary:

This is a retrospective cohort study of hybrid ML model that predicts postoperative AKI of adult patients who underwent major cardiac surgery using only preoperative information. The authors developed three AKI risk models and found that the hybrid ML showed the highest AUC among the models. It was an interesting subject. It seems that the authors have given sufficient answers to the comments and the feedback has been reflected in the paper. I think this paper deserves to be published in BMC Medical Research Methodology.

# Editor: Piero Lo Monaco - Accepted | 12-Apr-22

Thank you for addressing the last issues raised by the reviewers. Your manuscript is now ready for publication. I note that in a previous email exchange, you have agreed about transferring your manuscript to BMC Medical Informatics and Decision Making, where it will be accepted without any further review. Hence, your manuscript will be accepted here, but then our internal staff will withdraw it so that you can resubmit to BMC Medical Informatics and Decision Making. Please be informed that you will receive an email with more detailed instructions. Thank you for your understanding.
